# Pharmacological induction of AMFR increases functional EAAT2 oligomer levels and reduces epileptic seizures in mice

**DOI:** 10.1172/jci.insight.160247

**Published:** 2022-08-08

**Authors:** Longze Sha, Guanjun Li, Xiuneng Zhang, Yarong Lin, Yunjie Qiu, Yu Deng, Wanwan Zhu, Qi Xu

**Affiliations:** 1 State Key Laboratory of Medical Molecular Biology, Institute of Basic Medical Sciences Chinese Academy of Medical Sciences, School of Basic Medicine, Peking Union Medical College, Beijing, China.; 2 Neuroscience Center, Chinese Academy of Medical Sciences, Beijing, China.

**Keywords:** Neuroscience, Epilepsy

## Abstract

Dysregulation of excitatory amino acid transporter 2 (EAAT2) contributes to the development of temporal lobe epilepsy (TLE). Several strategies for increasing total EAAT2 levels have been proposed. However, the mechanism underlying the oligomeric assembly of EAAT2, impairment of which inhibits the formation of functional oligomers by EAAT2 monomers, is still poorly understood. In the present study, we identified E3 ubiquitin ligase AMFR as an EAAT2-interacting protein. AMFR specifically increased the level of EAAT2 oligomers rather than inducing protein degradation through K542-specific ubiquitination. By using tissues from humans with TLE and epilepsy model mice, we observed that AMFR and EAAT2 oligomer levels were simultaneously decreased in the hippocampus. Screening of 2386 FDA-approved drugs revealed that the most common analgesic/antipyretic medicine, acetaminophen (APAP), can induce AMFR transcriptional activation via transcription factor SP1. Administration of APAP protected against pentylenetetrazol-induced epileptogenesis. In mice with chronic epilepsy, APAP treatment partially reduced the occurrence of spontaneous seizures and greatly enhanced the antiepileptic effects of 17AAG, an Hsp90 inhibitor that upregulates total EAAT2 levels, when the 2 compounds were administered together. In summary, our studies reveal an essential role for AMFR in regulating the oligomeric state of EAAT2 and suggest that APAP can improve the efficacy of EAAT2-targeted antiepileptic treatments.

## Graphical abstract

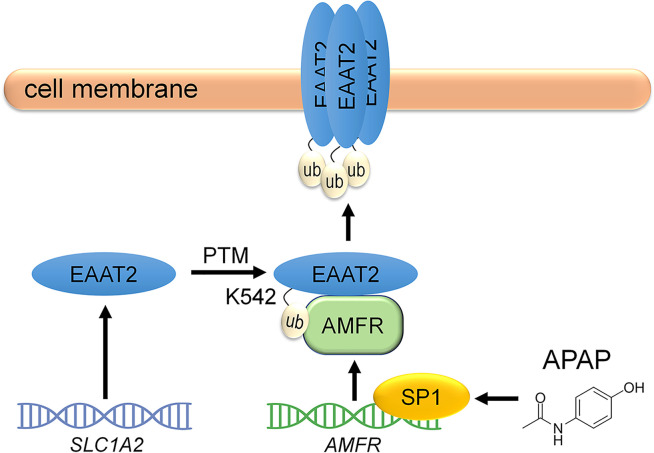

## Introduction

Epilepsy is one of the most common chronic neurological diseases; however, approximately one-third of affected patients, such as those with temporal lobe epilepsy (TLE), the most common form of medically refractory epilepsy, do not respond to antiepileptic drugs that were designed to modulate neuronal ion channels (1). Recent studies have shown that astrocytes may be a potential target for the treatment of TLE (2, 3). Astrocytes play a major role in maintaining physiological levels of interstitial glutamate. In the adult brain, excitatory amino acid transporter 2 (EAAT2; also known as GLT-1) is the major glutamate transporter, is expressed predominantly in astrocytes, and is responsible for the clearance of nearly 80% of interstitial glutamate (4). The dysregulation of EAAT2 leads to glutamate accumulation and excitotoxicity in TLE and many other neurological disorders, including amyotrophic lateral sclerosis (ALS), Alzheimer’s disease (AD), stroke, and Gulf War illness (5–8). Accordingly, it has been hypothesized that enhancing EAAT2 function could have antiexcitotoxic effects. Several strategies for increasing EAAT2 levels via transcriptional or translational activation have been proposed. Rothstein et al. first discovered that the β-lactam antibiotic ceftriaxone promotes EAAT2 expression but exerts a weak antiepileptic effect (9, 10). Kong et al. reported that a small molecule, LDN/OSU-0212320, increases EAAT2 expression through translational activation. LDN/OSU-0212320 reduces the frequency of spontaneous seizures in a mouse model of chronic TLE by approximately 50% (11). In addition, treatment with Hsp90 inhibitors, which prevents EAAT2 degradation, is also effective in reducing the occurrence of spontaneous seizures in mouse models (12, 13).

As a membrane-integrated transporter, EAAT2 needs to form a homotrimer to exert its glutamate clearance function. Posttranslational modifications (PTMs), including glycosylation, sumoylation, phosphorylation, and ubiquitination, regulate protein-protein interactions and ion channel assembly (14). However, the mechanism underlying the oligomeric assembly of EAAT2 through PTMs is largely unknown. It is worth mentioning that examinations of surgically resected hippocampal tissue from patients with TLE reveal that the level of total EAAT2 is not notably decreased in every sample (15), thus raising the possibility that the deficiency of functional EAAT2 oligomers (rather than the loss of EAAT2 monomers) may be involved in the accumulation of glutamate in some or even all patients.

AMFR is an E3 ubiquitin ligase anchored to the endoplasmic reticulum membrane (16). The functions of AMFR in the nervous system, especially in astrocytes, are still poorly understood. In this study, we found that AMFR interacts with EAAT2 and regulates the oligomeric state of EAAT2 through K542-specific ubiquitination. AMFR expression is downregulated in the sclerotic hippocampus in TLE patients and kainic acid–induced (KA-induced) epilepsy model mice. Genetic or pharmacological induction of AMFR expression has antiepileptic effects.

## Results

### AMFR is an EAAT2-interacting protein.

**Figure 1:**
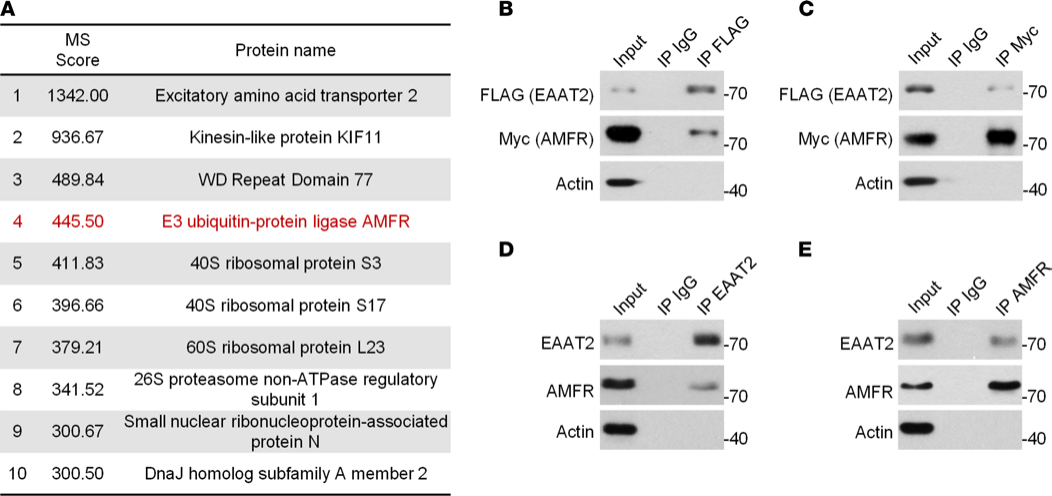
Identification of the interaction between EAAT2 and AMFR. (**A**) List of EAAT2-interacting proteins with a score value greater than 300 identified by MS. (**B**) Western blot analysis of EAAT2-FLAG after immunoprecipitation of AMFR-Myc. (**C**) Western blot analysis of AMFR-Myc after immunoprecipitation of EAAT2-FLAG. In **B** and **C**, HEK293 cells were cotransfected with EAAT2-FLAG and AMFR-Myc for 48 hours and lysed for immunoprecipitation. (**D**) Western blot analysis of immunoprecipitation of hippocampal lysates with an EAAT2-specific antibody followed by EAAT2 and AMFR immunoblotting. (**E**) Western blot analysis of immunoprecipitation of hippocampal lysates with an AMFR-specific antibody followed by AMFR and EAAT2 immunoblotting. In **B**–**E**, nonspecific rabbit IgG was used as a negative control. The blots are representative of 2 or 3 independent experiments. Numbers on the right of blots indicate kilodaltons.

To identify the molecules that participate in PTMs of EAAT2, FLAG-tagged EAAT2 and its binding proteins were immunoprecipitated from HEK293 cells, digested, and analyzed by using mass spectrometry (MS). Through MS, a total of 353 proteins were identified (Supplemental Data 1; supplemental material available online with this article; https://doi.org/10.1172/jci.insight.160247DS1), with 10 molecules having an ion score greater than 300. AMFR was the only protein-modifying enzyme (Figure 1A). AMFR is a RING finger E3 ubiquitin ligase. Successful detection of AMFR with its substrates by coimmunoprecipitation has been reported in many previous studies (16). The interaction between EAAT2 and AMFR was validated by using reciprocal immunoprecipitation and Western blotting in HEK293 cells that were transfected with epitope-tagged EAAT2 and AMFR (Figure 1, B and C) and in the mouse hippocampus (Figure 1, D and E).

### AMFR regulates the oligomeric state of EAAT2.

**Figure 2:**
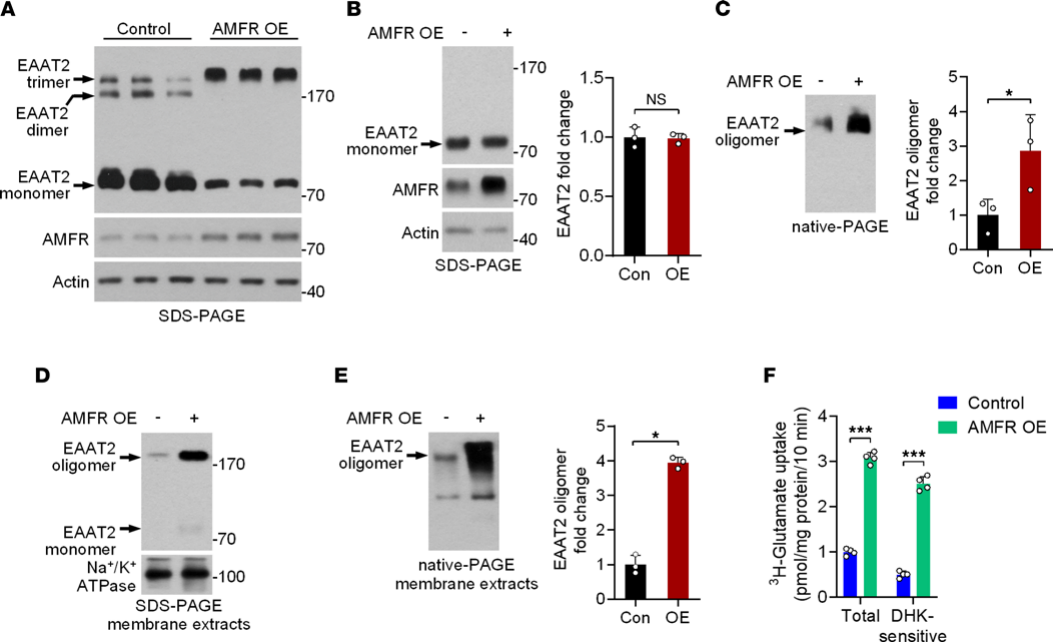
The effects of AMFR expression on EAAT2 oligomer levels. (**A**) SDS-PAGE followed by immunoblotting analysis of lysates of cultured astrocytes 48 hours posttransduction with AAV5-AMFR (OE) or control virus. (**B** and **C**) SDS-PAGE and native-PAGE followed by immunoblot analysis of lysates of cultured astrocytes 48 hours posttransduction of AAV5-AMFR or control virus. BME and DTT were used to promote the dissociation of EAAT2 oligomers in SDS-PAGE, except in **A**. The intensity of the EAAT2 bands was normalized to that of the Actin bands (*n* = 3). (**D** and **E**) SDS-PAGE and native-PAGE followed by immunoblot analysis of membrane extracts of cultured astrocytes 48 hours posttransduction of AAV5-AMFR or control virus. In **E**, the intensity of the EAAT2 oligomer bands was normalized to that of the Na^+^/K^+^ ATPase bands (*n* = 3). Numbers on the right of blots indicate kilodaltons. (**F**) Statistical analysis of ^3^H-glutamate uptake (*n* = 4). Cultured astrocytes were transduced with AAV5-AMFR or control virus for 72 hours, and DHK (100 μM), an EAAT2 inhibitor, was added 1 hour before the assay to distinguish DHK-sensitive glutamate uptake. OE, overexpression. Con, control. Student’s *t* test. *P < 0.05, ***P < 0.001.

A previous study reported that AMFR can promote the ubiquitin-mediated degradation of substrate proteins (17). However, we found that overexpression (OE) of AMFR had opposite effects on the levels of EAAT2 monomers and oligomers in primary cultured astrocytes subjected to denaturing electrophoresis (SDS-PAGE; Figure 2A). The EAAT2 oligomers increased as the monomers decreased. To better understand the effects of AMFR on EAAT2 monomers and oligomers, we completely promoted the dissociation of EAAT2 oligomers by using β-mercaptoethanol (BME) and dithiothreitol (DTT), and we again separated proteins by using SDS-PAGE. The results showed that OE of AMFR had no effect on the level of EAAT2 monomers, which represents total EAAT2 levels (SDS-PAGE; Figure 2B). Considering that ubiquitination also modulates protein-protein interactions, we examined the oligomeric state of EAAT2 by using nondenaturing electrophoresis (native-PAGE) and unexpectedly observed that EAAT2 oligomer levels were markedly increased in the AMFR OE group compared with the control group (Figure 2C). In contrast, knockdown (KD) of AMFR significantly decreased EAAT2 oligomer levels in primary cultured astrocytes (Supplemental Figure 1, A and B). Consistent with this finding, OE or KD of AMFR also regulated the level of EAAT2 oligomers in HEK293 cells (Supplemental Figure 1, C and D). Furthermore, we isolated the membrane fractions of primary cultured astrocytes. EAAT2 oligomers in the membrane fractions could not be fully denatured even in SDS-PAGE. The levels of both EAAT2 oligomers and monomers were increased when AMFR was overexpressed (Figure 2D). Native-PAGE analysis of the lysates of the membrane fractions specifically demonstrated an increase in the level of EAAT2 oligomers (Figure 2E).

EAAT2 oligomers are responsible for glutamate clearance. To explore whether OE of AMFR affects the uptake of glutamate by astrocytes, the uptake of ^3^H-glutamate by primary cultured astrocytes was assessed, and dihydrokainic acid (DHK), an EAAT2 inhibitor, was used to distinguish EAAT2-mediated glutamate uptake from glutamate uptake mediated by other glutamate transporters. The results showed that DHK-sensitive ^3^H-glutamate uptake activity was significantly increased in AMFR-overexpressing astrocytes (Figure 2F). Together, these data indicate an important role for AMFR in regulating the level of functional EAAT2. AMFR OE induced the formation of EAAT2 oligomers and promoted glutamate uptake by primary cultured astrocytes.

### AMFR specifically promotes ubiquitination of EAAT2 at K542.

**Figure 3:**
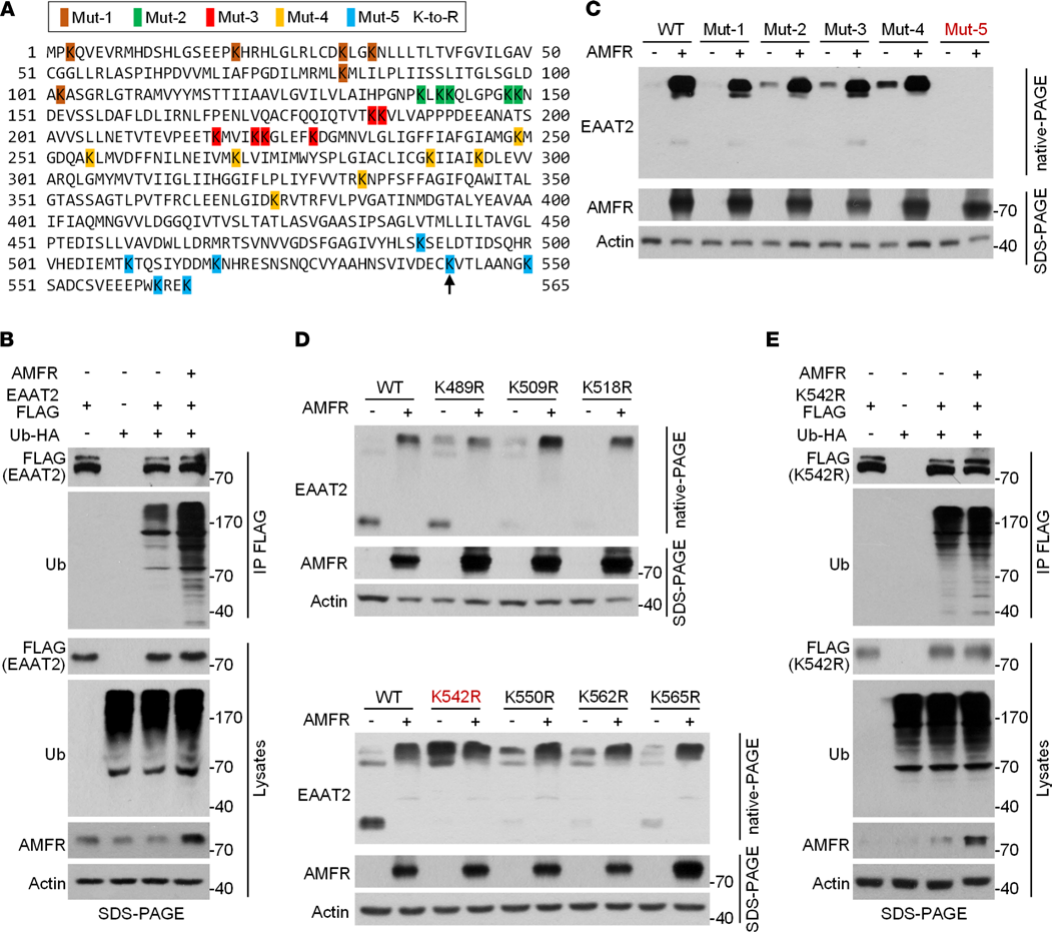
AMFR regulates the oligomeric state of EAAT2 through ubiquitination at K542. (**A**) Illustration of the EAAT2 K-to-R mutant constructs. The 31 lysine residues were divided into 5 groups, which are marked with different background colors. The arrow indicates the K542 site. (**B**) SDS-PAGE followed by immunoblot analysis of anti-FLAG–immunoprecipitated proteins from HEK293 cells transfected with AMFR, EAAT2-FLAG, Ub-HA, or the indicated combinations. (**C**) SDS-PAGE and native-PAGE followed by immunoblotting of HEK293 cells 48 hours posttransfection of expression vectors encoding WT/mutant EAAT2 and AMFR. Empty vector was used as a control for the AMFR vector. (**D**) SDS-PAGE and native-PAGE followed by immunoblot analysis of HEK293 cells 48 hours posttransfection of expression vectors encoding AMFR and WT or EAAT2 mutants, each of which harbored 1 of the 7 mutations in Mut-5. (**E**) Immunoblotting analysis of lysates of anti-FLAG-immunoprecipitated proteins from HEK293 cells transfected with AMFR, EAAT2 (K542R)-FLAG, Ub-HA, or the indicated combinations. Mut, mutant. The blots are representative of 2 or 3 independent experiments. Numbers on the right of blots indicate kilodaltons.

We next asked whether AMFR regulates the oligomeric state of EAAT2 via ubiquitination on lysine residues. The human EAAT2 protein contains 31 lysine residues (Figure 3A). We first demonstrated that OE of AMFR could increase the ubiquitination of EAAT2 (Figure 3B). To identify the site(s) of EAAT2 that were ubiquitinated by AMFR, we generated 5 EAAT2 K-to-R mutants, which each contained 5~7 arginine residues that replaced the original lysine residues (Figure 3A). In cotransfected HEK293 cells, the oligomer levels of the first 4 mutants (Mut-1 to Mut-4) exhibited a similar change in response to AMFR OE as WT EAAT2, whereas mutations in Mut-5 seemed to largely decrease the protein stability of EAAT2, and no EAAT2 band was detected regardless of whether AMFR was overexpressed (Figure 3C). Therefore, we further constructed 7 EAAT2 mutant plasmid vectors, each harboring 1 of the 7 K-to-R mutations in Mut-5. Interestingly, replacement of lysine with arginine at position 542 led to a marked increase in the level of EAAT2 oligomers compared with that of WT EAAT2, and the oligomeric state of the K542R mutant was not affected by AMFR OE (Figure 3D). The same result was true when lysine at position 542 was replaced by glutamate or glycine (Supplemental Figure 2). This result suggests that the lysine residue at position 542 prevents EAAT2 from forming oligomers and that ubiquitination of this residue could block this effect. As expected, ubiquitination of the EAAT2 K542R was not altered by AMFR OE (Figure 3E). Collectively, these data establish a specific role for AMFR-mediated ubiquitination of EAAT2 at K542 in increasing EAAT2 oligomer levels.

### AMFR expression is downregulated in tissues from humans with epilepsy and TLE model mice.

**Figure 4:**
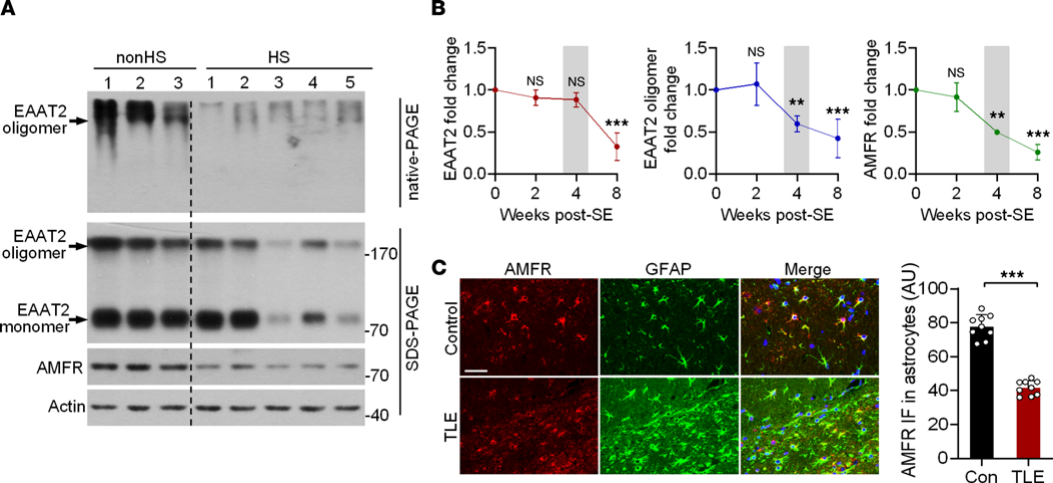
The expression of AMFR in the hippocampi of patients with TLE and KA-induced TLE model mice. (**A**) SDS-PAGE and native-PAGE followed by immunoblot of lysates of non-HS and HS samples. (**B**) Statistical analysis of EAAT2, AMFR (SDS-PAGE), and EAAT2 oligomer (native-PAGE) levels in the sclerotic hippocampi of mice 0–8 weeks after KA-induced SE. Western blots are shown in Supplemental Figure 2. Numbers on the right of blots indicate kilodaltons. (**C**) IF staining of AMFR (red) and GFAP (green) and DAPI staining (blue) in the sclerotic hippocampi of mice 4 weeks after KA-induced SE and in the hippocampi of saline-injected control mice (left). Quantification of the AMFR IF intensity in GFAP-labeled astrocytes (right; *n* = 9 slices from 3 mice). Scale bar: 20 μm. Student’s *t* test (**B** and **C**). **P < 0.01, ***P < 0.001.

Given that AMFR regulates the oligomeric state of EAAT2, we sought to examine whether this mechanism is disrupted in TLE. We first examined EAAT2 levels in 3 control non–hippocampal sclerosis (non-HS) samples and 5 hippocampal sclerosis (HS) samples from patients with TLE using both denaturing and nondenaturing electrophoresis. After SDS-PAGE, most EAAT2 proteins were denatured and presented as monomers (Figure 4A). The expression of total EAAT2 was downregulated in 3 of the 5 HS samples (Nos. 3–5). Interestingly, although the total EAAT2 levels in samples No. 1 and No. 2 from the HS group were comparable to those in the non-HS group (SDS-PAGE in Figure 4A), the level of EAAT2 oligomers was lower in these samples (native-PAGE in Figure 4A), possibly due to decreased AMFR levels (Figure 4A). Indeed, compared with the 3 non-HS samples, all 5 HS samples exhibited a consistent reduction in the level of AMFR (Figure 4A). Having found that AMFR expression is downregulated in the hippocampi of patients with TLE, we looked for a similar pattern in the hippocampus of the KA-induced post–status epilepticus (post-SE) mouse model of TLE. In the post-SE model of TLE, the level of total EAAT2 in the hippocampus ipsilateral to KA injection began to decrease until the eighth week post-SE (Figure 4B and Supplemental Figure 3). However, the level of EAAT2 oligomers was already decreased at 4 weeks post-SE, and this effect was accompanied by downregulation of AMFR expression (Figure 4B and Supplemental Figure 3). By using immunofluorescence (IF), the decrease in the level of AMFR was further validated in glial fibrillary acidic protein–labeled (GFAP-labeled) hippocampal astrocytes (Figure 4C). Collectively, our results suggest that EAAT2 oligomer levels are reduced prior to the decrease in total EAAT2 protein levels during TLE and that the early reduction in AMFR levels may be responsible for the loss of functional EAAT2 oligomers in the epileptic hippocampus.

### Hippocampal expression of AMFR exerts antiepileptic effects in the acute seizure model.

**Figure 5:**
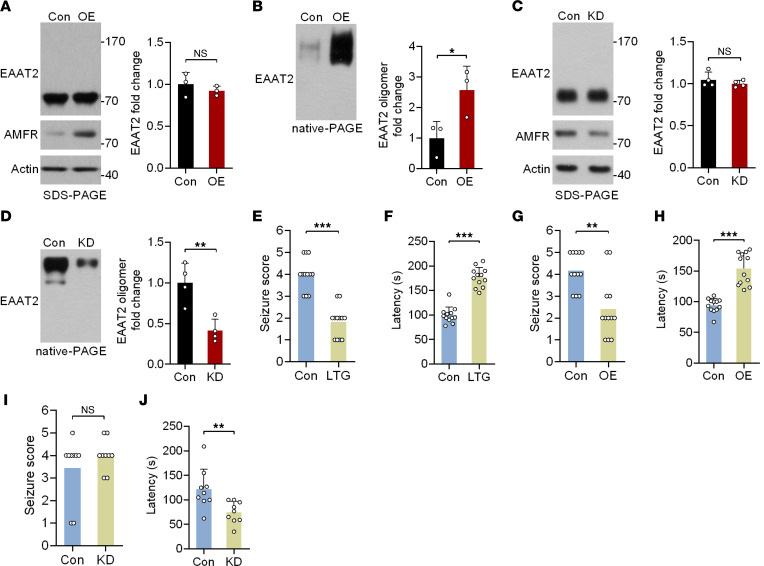
The antiepileptic effects of AMFR expression in the pentylenetetrazol-induced acute seizure mouse model. (**A** and **B**) SDS-PAGE and native-PAGE followed by immunoblotting of lysates from the hippocampi of mice at 4 weeks after hippocampal injection of AAV5-AMFR OE or control virus (*n* = 3). (**C** and **D**) SDS-PAGE and native-PAGE followed by immunoblotting analysis of lysates from the hippocampi of mice at 4 weeks after hippocampal injection of AAV-AMFR KD or control virus (*n* = 4). Numbers on the right of blots indicate kilodaltons. (**E**–**J**) Statistical analyses of the seizure score and latency of the acute response to PTZ. (**E** and **F**) Mice were administered LTG (20 mg/kg) or vehicle 1 hour before PTZ injections. (*n* = 12). (**G** and **H**) Mice were studied 4 weeks after bilateral injection of AAV5-AMFR OE or control virus (*n* = 12). (**I** and **J**) Mice were studied 4 weeks after bilateral injection of AAV5-AMFR KD or control virus. (*n* = 9.) Student’s *t* test (**A**–**J**). *P < 0.05, **P < 0.01, ***P < 0.001.

Given the potential role of AMFR in regulating the oligomeric state of EAAT2, we asked whether upregulation of AMFR expression has antiepileptic activity. Consistent with the aforementioned in vitro results, OE of AMFR in the mouse hippocampus did not affect total EAAT2 levels but markedly increased EAAT2 oligomer levels (Figure 5, A and B). Conversely, KD of AMFR led to decreased levels of EAAT2 oligomers (Figure 5, C and D). Next, we asked whether the interference with AMFR expression affects pentylenetetrazol-induced (PTZ-induced) acute seizures. In the PTZ model, pretreatment with the positive control lamotrigine (LTG) significantly decreased the severity of acute seizures (Figure 5E). Moreover, mice in the LTG group showed a delayed response to PTZ (Figure 5F). Similar to LTG, OE of AMFR in the bilateral hippocampus also exerted a protective effect against PTZ-induced acute seizures (Figure 5, G and H). Conversely, although KD of AMFR did not exacerbate seizures (Figure 5I), the latency of the response to PTZ was significantly shorter in AMFR-KD mice than in control mice (Figure 5J).

### Identification of acetaminophen as an AMFR inducer.

**Figure 6:**
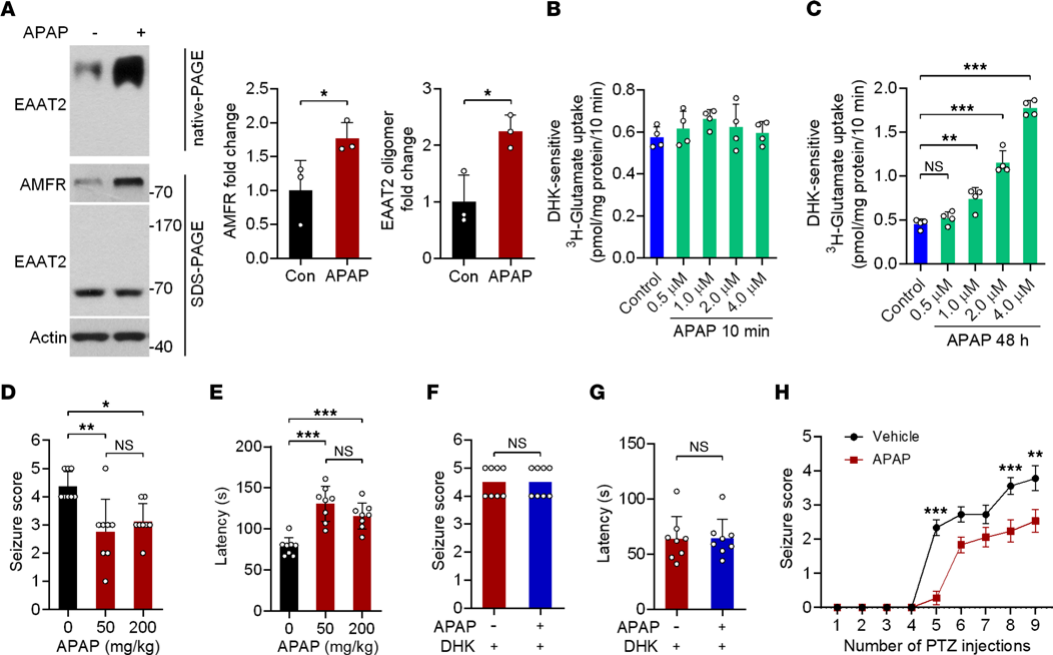
APAP upregulates AMFR protein expression and exhibits antiepileptic effects. (**A**) SDS-PAGE and native-PAGE followed by immunoblotting analysis of lysates from the hippocampus ipsilateral to KA injection (left). Five weeks after hippocampal injection of KA, mice were given 50 mg/kg APAP or vehicle for 5 days. Statistical analyses of AMFR levels in samples subjected to SDS-PAGE and EAAT2 oligomer levels in samples subjected to native-PAGE (right). Numbers on the right of blots indicate kilodaltons. (**B** and **C**) Statistical analyses of DHK-sensitive ^3^H-glutamate uptake (*n* = 4). Cultured astrocytes were treated with 0.5 to 4 μM APAP or vehicle for 10 minutes or 48 hours, and DHK (100 μM) was added 1 hour before the assays to distinguish DHK-sensitive glutamate uptake. (**D** and **E**) Acute model of epilepsy. Statistical analyses of the difference in seizure scores and latency of the acute response to PTZ in vehicle control– and APAP-pretreated mice at 50 or 200 mg/kg (*n* = 8). (**F** and **G**) PTZ-induced model of acute seizure. Statistical analyses of the difference in seizure scores and latency of the acute response to PTZ in APAP-pretreated (50 mg/kg) and APAP/DHK-pretreated mice (*n* = 8). DHK (10 mg/kg) was i.p. injected 30 minutes before PTZ. (**H**) Statistical analyses of the difference in seizure scores in the PTZ kindling model of epileptogenesis (*n* = 18). Student’s *t* test (**A**, **F**, **G**). One-way ANOVA followed by Dunnett’s post hoc test (**B**–**E**). Two-way ANOVA followed by post hoc multiple comparisons test (**H**). *P < 0.05, **P < 0.01, ***P < 0.001.

The above studies suggested that restoring EAAT2 oligomer levels by increasing AMFR levels may represent an effective strategy for treating TLE. To test this possibility, we screened 2386 FDA-approved drugs using an in-cell ELISA and identified 5 compounds that can upregulate AMFR protein expression, including acetaminophen (APAP), adapalene, tolcapone, resveratrol, and retinoic acid (Supplemental Figure 4A). The upregulating effect of the 5 compounds on AMFR protein expression was further validated in primary cultured astrocytes by Western blotting (Supplemental Figure 4B). Normal mice were injected with each of the 5 drugs, but only APAP significantly increased AMFR protein levels in the hippocampus (Supplemental Figure 4, C–H). In normal mice, the AMFR levels were increased at 50 mg/kg. Although the dose of 200 mg/kg induced more AMFR proteins than 50 mg/kg, it did not further increase EAAT2 oligomers (Supplemental Figure 4H). The injection of APAP at 50 mg/kg also increased EAAT2 oligomer levels in the ipsilateral (KA-injected) hippocampus of both male and female mice at 5 weeks post-SE (Figure 6A and Supplemental Figure 5A). Similar to AMFR OE, chronic (but not acute) APAP treatment promoted DHK-sensitive, EAAT2-mediated glutamate uptake by primary cultured astrocytes in a dose-dependent manner (Figure 6, B and C).

Given the role of APAP in increasing EAAT2 oligomer levels, we assessed the pharmacological effects of APAP on a PTZ-induced acute epilepsy model. Daily injections of 50 mg/kg or 200 mg/kg APAP for 3 days prior to PTZ administration decreased the severity of seizures in mice. The average seizure score in response to challenge with 55 mg/kg PTZ in the APAP group was lower than that in the control group (Figure 6D). APAP-treated mice showed a delayed response to PTZ (Figure 6E). Consistent with the result that the dose of 50 mg/kg was sufficient to induce the largest number of EAAT2 oligomers (Supplemental Figure 4G), the antiepileptic effects of APAP reached a maximal level at the same dose (Figure 6, D and E). In addition, DHK completely abolished the antiepileptic effects of APAP (Figure 6, F and G).

To examine whether APAP treatment affects epileptogenesis, mice were repeatedly injected with 40 mg/kg PTZ. In the vehicle-injected control group, PTZ kindling increased seizure severity after 9 PTZ injections, while simultaneous treatment with APAP significantly decreased the average seizure score (Figure 6H).

### APAP promotes AMFR transcription via transcription factor SP1.

**Figure 7:**
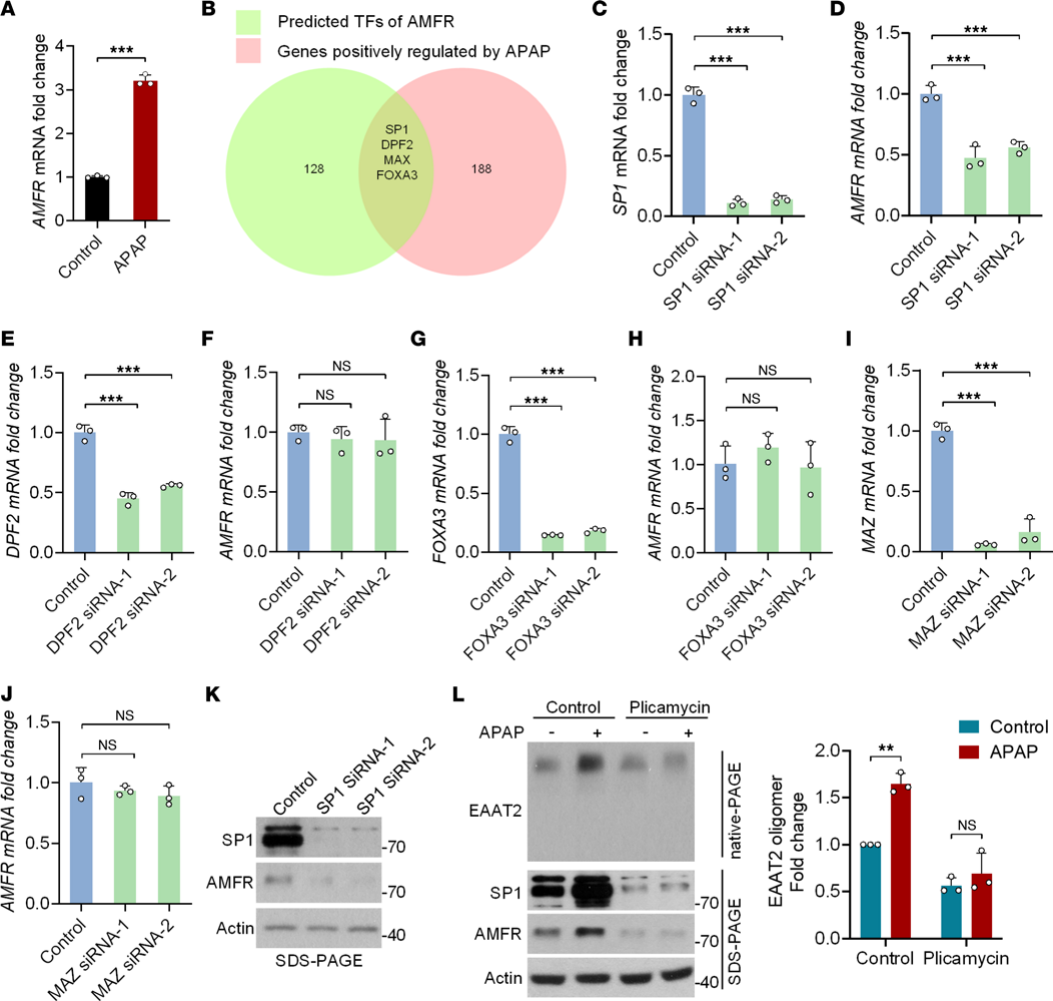
APAP upregulates AMFR protein expression through the transcription factor SP1. (**A**) Quantitative PCR and statistical analysis of the mRNA level of *AMFR* in primary cultured astrocytes 24 hours after APAP treatment (2 μM). (**B**) The transcription factors (TFs) predicted to upregulate AMFR expression (green, 128 genes) and APAP-regulated TFs (red, 188 genes). The shared TFs were DPF2, FOXA3, MAZ, and SP1. (**C** and **D**) Quantitative PCR and statistical analysis of *SP1* and *AMFR* mRNA levels in primary cultured astrocytes 48 hours after transfection with 2 independent siRNAs targeting SP1 (*n* = 3). (**E** and **F**) Quantitative PCR and statistical analysis of *DPF2* and *AMFR* mRNA levels in primary cultured astrocytes 48 hours after transfection with 2 independent siRNAs targeting DPF2 (*n* = 3). (**G** and **H**) Quantitative PCR and statistical analysis of *FOXA3* and *AMFR* mRNA levels in primary cultured astrocytes 48 hours after transfection with 2 independent siRNAs targeting FOXA3 (*n* = 3). (**I** and **J**) Quantitative PCR and statistical analysis of *MAZ* and *AMFR* mRNA levels in primary cultured astrocytes 48 hours after transfection with 2 independent siRNAs targeting MAZ (*n* = 3). (**K**) Immunoblot analysis of primary cultured astrocytes 48 hours after transfection with 2 independent siRNAs targeting SP1. (**L**) Western blot analysis of primary cultured astrocytes 48 hours after treatment with the SP1 inhibitor plicamycin (1 μM), APAP (2 μM), or their combination as indicated. Numbers on the right of blots indicate kilodaltons. Student’s *t* test (**A** and **L**). One-way ANOVA followed by Dunnett’s post hoc test (**C**–**J**). **P < 0.01, ***P < 0.001.

Quantitative real-time PCR confirmed that APAP upregulated AMFR protein expression via transcriptional activation (Figure 7A). To identify the specific transcription factor(s) (TF) responsible for APAP-induced AMFR expression, we compared the TFs of AMFR predicted by GeneHancer (GH16J056420) with a data set related to the APAP-induced global transcriptional response (18) and identified 4 overlapping TFs (SP1, DPF2, FOXA3, and MAZ) (Figure 7B). KD of SP1 in primary cultured astrocytes significantly decreased the mRNA level of *AMFR* (Figure 7, C and D), while KD of the other 3 TFs had no such effect (Figure 7, E–J). Moreover, the protein level of AMFR was decreased when SP1 expression was downregulated (Figure 7K). In addition, the SP1 inhibitor plicamycin completely abolished the ability of APAP to increase both AMFR protein levels and EAAT2 oligomer levels (Figure 7L), suggesting that SP1 is necessary for APAP-induced AMFR transcriptional activation.

To determine whether the ability of APAP to increase EAAT2 oligomer levels is due to enhancement of oligomeric assembly or inhibition of dissociation, we used Sulfo-NHS-SS-Biotin to label EAAT2 oligomers in the cell membranes of cultured astrocytes and examined EAAT2 oligomer levels 12 hours and 24 hours after APAP or vehicle treatment. As shown in Supplemental Figure 5B, there was no difference in EAAT2 oligomer levels between the APAP and vehicle groups, thus implying that APAP promoted oligomeric assembly rather than inhibiting the dissociation of EAAT2.

### APAP reduces chronic seizures in the KA-induced TLE mouse model.

**Figure 8:**
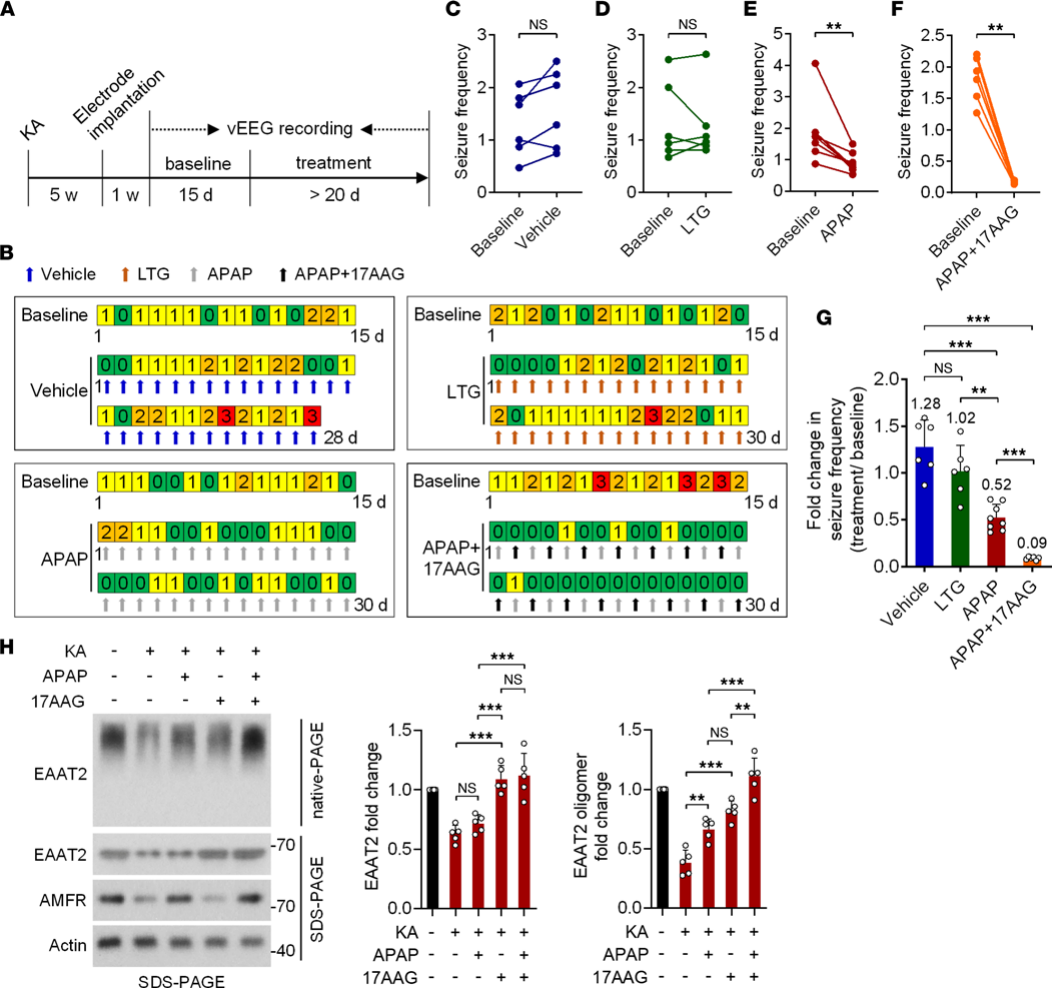
The antiepileptic effects of APAP in a mouse model of chronic TLE. (**A**) Experimental design. (**B**) Examples of daily recordings of spontaneous recurrent seizures in the vehicle, LTG, APAP, and APAP/17AAG treatment groups. LTG was administered i.p. at a dose of 20 mg/kg daily (*n* = 6). APAP was administered i.p. at a dose of 50 mg/kg daily (*n* = 8). 17AAG was administered i.p. at a dose of 25 mg/kg once every other day (*n* = 6). DMSO was used as a vehicle control (*n* = 6). (**C**–**F**) Statistical analyses of the difference in seizure frequency (average number of seizures per day) between baseline and after treatment. (**G**) Statistical analysis of the difference in the fold change in seizure frequency before and after treatment with vehicle, LTG, 17AAG, or APAP/17AAG. (**H**) Native-PAGE and SDS-PAGE followed by immunoblotting analysis of lysates from the hippocampus ipsilateral to KA injection (left; *n* = 5). Five weeks after hippocampal injection of KA, mice were given APAP, 17AAG, or their combination for 2 weeks. Statistical analysis of the level of total EAAT2 in samples subjected to SDS-PAGE and EAAT2 oligomer levels in samples subjected to native-PAGE (right). Numbers on the right of blots indicate kilodaltons. Paired *t* test (**C**–**F**). Student’s *t* test (**G**). Two-way ANOVA followed by post hoc multiple comparisons test (**H**). **P < 0.01, ***P < 0.001.

Subsequently, by using the KA-induced post-SE model of TLE, we investigated whether APAP has antiepileptic activity in mice that have already developed spontaneous seizures. Figure 8, A and B, show the experimental procedures. In the vehicle control group, the seizure frequency was increased 1.28-fold after administration of DMSO compared with before DMSO administration (Figure 8C). The intrahippocampal injection of KA reliably models refractory TLE with HS, and the model mice displayed drug-resistant characteristics after repeated administration of LTG (Figure 8D). Moreover, once-daily treatment with APAP significantly suppressed the occurrence of spontaneous seizures (50 mg/kg; Figure 8E), but the seizure frequency was only decreased by about 50% compared with baseline (Figure 8G). We speculated that this may have been because the level of total EAAT2 was already decreased in the epileptic focus, as observed in the HS samples (Figure 4A). Our previous study demonstrated that the Hsp90 inhibitor 17AAG can upregulate the expression of total EAAT2 by preventing excessive degradation of EAAT2 in the epileptic hippocampus (12). Therefore, we asked whether combined treatment with 17AAG and APAP exerts stronger antiepileptic effects by simultaneously upregulating the protein level of total EAAT2 and by restoring the oligomeric assembly of EAAT2. As expected, cotreatment with the 2 compounds dramatically inhibited the occurrence of spontaneous seizures by 91%, exerting a stronger effect than APAP (48%; Figure 8, F and G) or 17AAG monotherapy (73%; as reported in our previous study, ref. 12). In addition, APAP/17AAG polytherapy led to a more significant decline in the incidence of interictal epileptiform discharges than APAP monotherapy (Supplemental Figure 5, C–G). Consistent with the EEG results, examinations of the levels of total EAAT2 and EAAT2 oligomers in the epileptic hippocampus demonstrated that APAP/17AAG polytherapy could increase EAAT2 oligomer levels to a maximum value when compared with 17AAG treatment alone, although it could not further elevate the level of total EAAT2 (Figure 8H). In summary, these findings suggest that APAP exhibits antiepileptic effects in mice with chronic spontaneous epilepsy. Restoring oligomeric state of EAAT2 through induction of AMFR could significantly improve the efficiency of EAAT2-targeted antiepileptic treatments.

## Discussion

In this study, we demonstrated that AMFR interacted with EAAT2 and specifically regulated the level of EAAT2 oligomers rather than inducing protein degradation through K542-specific ubiquitination. The most common analgesic/antipyretic medicine, APAP, can induce AMFR transcriptional activation via SP1 and exerted an antiepileptic effect in mouse models of epilepsy.

### The role of ubiquitination in regulating EAAT2 function.

Although the canonical role of ubiquitination is to mediate the degradation of proteins tagged with a single or polymeric ubiquitin chain by the proteasome, some studies have reported that ubiquitination can also promote or inhibit protein interactions rather than promoting degradation (19). Both monoubiquitination and polyubiquitination are involved in this process. AMFR is a membrane-bound E3 ubiquitin ligase embedded in the endoplasmic reticulum that plays an important role in endoplasmic reticulum–associated degradation. In this study, we reported that AMFR may participate in the regulation of the oligomeric assembly of EAAT2 through site-specific ubiquitination. The cytosolic carboxy-terminal domain is critical for modulating the function of EAAT2. Different lysine residues within this fragment have their own functions. Ubiquitination of EAAT2 at K509 or K518 promotes EAAT2 degradation (20). The present study found that ubiquitination at K542 facilitates EAAT2 polymerization. Interestingly, the lysine residue at position 542 seems to prevent oligomeric assembly of EAAT2. Lysine is a positively charged basic amino acid that plays important roles in protein folding by forming electrostatic interactions (21). Ubiquitination of K542 may change the local spatial arrangement of the C-terminus of EAAT2, thus making EAAT2 monomers more prone to interact with each other. This is reasonable because many ion channels and transporters exhibit a self-assembling tendency (22), and it is necessary to prevent the self-assembly of immature monomers. Ubiquitination-mediated oligomeric assembly is likely a step of quality control for protein folding in the endoplasmic reticulum (23); however, to our knowledge, there have been no studies on this topic. In addition, ubiquitination is a reversible modification. Whether deubiquitination at K542 promotes dissociation of EAAT2 oligomers anchored to the cell membrane merits further investigation.

### Distinct mechanisms of EAAT2 deficiency in different stages of epileptogenesis.

It has been widely accepted that the dysregulation of EAAT2 is a primary mechanism of temporal lobe epileptogenesis (2, 24). The restoration of the glutamate clearance function of EAAT2 has been recognized as a promising way to treat excitotoxicity-related disorders, especially TLE. The development of TLE can be divided into the following 3 stages: a) the initial brain lesion, b) the latent period, and c) the chronic epilepsy phase (25). The mechanisms underlying EAAT2 deficiency seem to be different in each stage. To achieve the maximum therapeutic effect, antiepileptic treatment should be mechanism directed. The failure to address the mechanism underlying the molecular deficiency of EAAT2 is expected to result in poor antiepileptic efficacy. For example, the EAAT2 transcriptional activator ceftriaxone is effective in alleviating epileptogenesis when administered beginning in the early stage (26). In the chronic stage of TLE, excessive protein degradation is the primary reason for the loss of total EAAT2 levels; therefore, ceftriaxone has no significant inhibitory effect on chronic spontaneous seizures in epilepsy model mice (12). The present study suggests that downregulation of AMFR expression in the latent and chronic stages impairs oligomerization of EAAT2, which suggests that the induction of AMFR expression should be considered an important strategy for treating epilepsy in both stages.

### The antiepileptic activity of APAP.

In this proof-of-concept study, we screened 2386 FDA-approved drugs and identified APAP as an AMFR inducer. APAP could increase EAAT2 oligomer levels through transcriptional activation of AMFR and exert antiepileptic effects. APAP is a nonsteroidal antiinflammatory drug that exhibits both analgesic and antipyretic properties and has been widely used as an active ingredient in many approved drugs. Several human and animal studies have found that APAP has protective effects against acute seizure, but the explanations of the underlying mechanisms are different. In a clinical trial, APAP was found to prevent febrile seizure recurrence during the same fever episode (27). The authors believed that APAP may reduce febrile seizure recurrence through effects other than antipyretic effects because: a) the body temperature was not different between the APAP and control groups and b) a previous clinical trial examined the overall effect of 4 types of antipyretics and found that they are ineffective for the prevention of recurrences of febrile seizures (28). In an animal study, APAP was found to exert antiepileptic effects in PTZ-kindled mice at a high dose (350 mg/kg), and the mechanism did not involve prostanoid signaling cascades (which is the primary pharmacological target of APAP), due to the fact that 3 other nonsteroidal antiinflammatory drugs had no such effect (29). AM404 is an active metabolite of APAP. It has been shown to exhibit antiepileptic activity in several animal and cellular models of acute seizure. The beneficial effects of AM404 have been attributed to the activation of endocannabinoid and the transient receptor potential vanilloid-1 systems (30–32). Our results suggest that APAP induces a potentially novel SP1/AMFR/EAAT2 cascade in astrocytes and exerts antiepileptic effects in mice with chronic TLE at a lower dose compared with the above studies (50 mg/kg vs. 350 mg/kg). The maximum tolerated dose of APAP in mice was approximately 350–400 mg/kg. In human adults, acute ingestion of more than 150 mg/kg APAP is toxic. In this study, the i.p. administration of 50 mg/kg APAP, which is equivalent to 10 mg/kg (p.o.) in humans based on the guide for dose conversion between animals and humans and the difference in bioavailability between oral and intravenous administrations of APAP (33, 34), was sufficient to produce antiepileptic effects in the TLE mouse model. However, it is unclear whether the long-term administration of APAP to treat TLE, even at low doses, is safe for humans; thus this question merits further investigation. Furthermore, APAP can increase serotonin levels in the brain, which may counteract its antiepileptic effect (35, 36). Collectively, the antiepileptic effects of APAP in animal models of epilepsy may result from the net change in multiple neurotransmitters by modulating both neurons and astrocytes.

It is worth noting that missense mutations in the critical domain of EAAT2 are able to change the direction of glutamate flow, i.e., G82R and L85P in EAAT2 (37), which suggests that genetic testing should be performed prior to EAAT2-based therapy in patients with epilepsy to avoid more severe excitotoxicity.

In summary, this study demonstrated the therapeutic value of AMFR induction for TLE. Considering that glutamate excitotoxicity is a common mechanism of neuronal injury that is implicated in the pathogenesis of many neurological disorders, including ALS, AD, and stroke, whether AMFR deficiency is involved in the pathogenesis of these diseases deserves further investigation.

## Methods

### Human tissues.

Two neuropathologists reviewed all cases. In 5 patients recruited from Peking Union Medical College Hospital with TLE (Nos. 1–5), HS without extrahippocampal pathology was identified. The control non-HS samples were obtained from 3 patients diagnosed with gliomas (No. 1) or cortical dysplasia (Nos. 2 and 3) that did not involve the hippocampus proper, and the histological examination demonstrated no significant hippocampal neuronal loss in these patients.

### Animals.

Male mice were used in this study, unless otherwise specified. Adult *C57BL/6J* mice were purchased from SPF (Beijing) Biotechnology Co., Ltd. Adult *C57BL/6J* mice were housed in groups. Temperature and light were controlled throughout the experiment. All the mice had free access to standard chow and water. For wired EEG recordings, mice were individually housed and monitored daily.

### Cells and immunoprecipitation analysis.

All cell lines were obtained from Cell Resource Center of the Institute of Basic Medicine Sciences Chinese Academy of Medical Sciences (IBMS & CAMS, Beijing, China). HEK293 cells were washed once with cold PBS and solubilized on ice in CelLytic M lysis buffer (C2978; Sigma-Aldrich) supplemented with protease inhibitors (100×, B14001; Bimake). Cleared lysates were harvested by centrifugation at 13,000*g* for 15 minutes at 4°C, and 1.0 mg of each lysate was used for immunoprecipitation. The lysates were incubated with EZview Red Anti-FLAG Agarose Beads Affinity Gel (F2426; Sigma-Aldrich) or EZview Red Anti-c-Myc Agarose Beads Affinity Gel (E6654; Sigma-Aldrich) and isotype control IgG antibody at 4°C with gentle rotation for 4 hours. After 3 washes with PBS, the immunoprecipitated proteins were recovered from the beads by boiling for 10 minutes in sample buffer. Rabbit anti-EAAT2 antibody (22515-1-AP; Proteintech) and rabbit anti-AMFR antibody (16675-1-AP; Proteintech) were used for in vivo coimmunoprecipitation from mouse hippocampal tissues, and immunoprecipitated proteins were detected with mouse anti-EAAT2 antibody (sc-365634; Santa Cruz Biotechnology) and mouse anti-AMFR antibody (sc-166358; Santa Cruz Biotechnology).

### MS.

EAAT2-FLAG recombinant proteins were purified from HEK293 cells. The recovered proteins were separated by SDS-PAGE, and then the digested peptides were analyzed by MS (conducted by the Center for Mass Spectrometry, Tsinghua University, Beijing, China) to identify proteins that were captured by the FLAG antibody. According to the MS results, proteins with an ion score greater than 300 in the experimental group were considered high-confidence interacting proteins.

### Primary culture of astrocytes.

Primary astrocytes were isolated from newborn (12–24 hours after birth) *C57BL/6J* pups as previously described. Cerebral cortex tissues were obtained from newborn *C57BL/6J* pups, and the meninges and blood vessels were removed. The remaining tissue was mechanically dissociated with a 25-gauge needle and suspended in DMEM (CM15019; Macgene) containing 20% FBS (10099141; Gibco) and 20 ng/mL EGF (315-09; PeproTech). Dispersed cells were seeded in T25 culture flasks coated with poly-d-lysine (PDL, P6407; Sigma-Aldrich) and maintained in a humid atmosphere containing CO_2_/air (5%/95%) at 37°C for 7 to 10 days without changing the culture medium. Microglia and oligodendrocytes were removed by placing the flasks in a heated shaker (37°C) overnight after the cells reached confluence. Astrocytes were then replated in PDL-coated dishes for subsequent experiments.

### Plasmid constructs and transfection.

EAAT2-FLAG, Ub-HA, and AMFR-Myc were subcloned into the pcDNA3.1 vector (V855-20; Invitrogen, Thermo Fisher Scientific). The EAAT2 K-to-R mutants were synthesized by Tsingke. Neofect DNA transfection reagent was used for transient transfection of the plasmids. All the siRNAs, i.e., AMFR siRNA-1 (5′-GACCTCGCTTAAACCAACA-3′) and AMFR siRNA-2 (5′-GCAGAATGTCTCTTAATAT-3′) for humans and AMFR siRNA-1 (5′-GGACTTCAGTGAGGTAGAA-3′), AMFR siRNA-2 (5′-GGTCATCTTTATGCAACTT-3′), SP1 siRNA-1 (5′-GCAGGATGGTTCTGGTCAA-3′), SP1 siRNA-2 (5′-CAGACTAGCAGCAGCAGTAATA-3′), MAZ siRNA-1 (5′-CGACATAAGCTGTCGCATT-3′), MAZ siRNA-2 (5′-GCATCTGTCTTGGAGAAGA-3′), Fox3A siRNA-1 (5′-GCATTCGCTGTCCTTCAAT-3′), Fox3A siRNA-2 (5′-GCAGTTAGACTGGTGTACT-3′), DPF2 siRNA-1 (5′-GCTGGACAGTTTGTCTCTT-3′), and DPF2 siRNA-2 (5′-GCGAGTTTCCTGTTAGCAA-3′) for mice, were synthesized by RiboBio. Lipofectamine RNAiMAX (13778030; Thermo Fisher Scientific) was used for siRNA transfection.

### Protein electrophoresis and Western blotting.

CelLytic M was used to extract protein from cultured cells and brain tissues. Membrane proteins were extracted using the Mem-PER Plus Kit (89842; Thermo Fisher Scientific). Protein samples prepared for SDS-PAGE analysis were denatured by heating in the presence of loading buffer containing 2% SDS (20 mM BME and 100 mM DTT, except in Figure 2A) and separated on a handmade SDS-PAGE gel. Protein samples used for native-PAGE were prepared in loading buffer containing 0.1% SDS (but not BME and DTT) to reduce the dispersion of the EAAT2 bands, after which they were separated on a precast 12% native-PAGE gel (PG01210-N; Solarbio). The following antibodies were used: mouse anti–β-Actin (66009-1-Ig) and rabbit anti-EAAT2 (22515-1-AP), anti-SP1 (21962-1-AP), anti-ubiquitin (10201-2-AP), and anti-AMFR (16675-1-AP) from Proteintech; mouse anti-EAAT2 (sc-365634) from Santa Cruz Biotechnology; and mouse monoclonal anti-FLAG M2 (F1804) from Sigma-Aldrich.

### Sulfo-NHS-SS-Biotin labeling experiment.

To label the membrane proteins, the cells were washed 3 times with PBS, after which they were incubated with 1 mg/mL EZ-Link Sulfo-NHS-SS-Biotin (21331; Thermo Fisher Scientific) in PBS for 30 minutes at 37°C. The cells were then washed 3 times with quenching buffer (25 mM Tris in PBS) to remove any nonreacted biotinylation reagent. After labeling with Sulfo-NHS-SS-Biotin, cells were cultured in DMSO or APAP (2 μM) for 12 or 24 hours, respectively, and harvested for the assessment of biotin-labeled protein by incubation with NeutrAvidin Agarose Resins (29201; Thermo Fisher Scientific) overnight at 4°C. The next day, after 3 rinses with PBS, the biotin-labeled proteins were recovered from the agarose by boiling for 10 minutes in sample buffer, after which they were used for Western blotting analysis.

### Analysis of ^3^H-glutamate uptake by cultured astrocytes.

Cultured astrocytes in 24-well plates were washed twice and then incubated in Krebs buffer (135 mM NaCl, 5 mM KCl, 0.6 mM MgSO_4_, 1 mM CaCl_2_, 6 mM d-glucose, and 10 mM HEPES; pH 7.5) containing 20 nM ^3^H-glutamate (0.5 μCi; specific activity = 50.8 Ci/mmol; PerkinElmer) and 40 μM unlabeled glutamate for 10 minutes at 37°C. DHK was used to distinguish DHK-sensitive (EAAT2) ^3^H-glutamate uptake from DHK-insensitive uptake. After incubation, the Krebs buffer was replaced with PBS to stop the uptake of glutamate, and the cells were then collected in cell lysis solution (C2978; Sigma-Aldrich). The lysates were processed to measure both ^3^H-glutamate levels with a liquid scintillation counter (LS6500 scintillation counter; Beckman Coulter) and protein content by using the Bradford Protein Assay kit (5000002; Bio-Rad). The concentration of glutamate taken up by the astrocytes (in nanomolars) was calculated using the radioactivity and the total protein concentration per milligram of protein over a 10-minute period. DHK-sensitive ^3^H-glutamate uptake was calculated as the total amount of Na^+^ dependent ^3^H-glutamate taken up minus the amount of DHK-insensitive Na^+^-dependent ^3^H-glutamate taken up (DHK-treated samples). All values were normalized to mean glutamate uptake in the control group, and all measurements were repeated 3 times.

### Quantitative real-time PCR.

Total RNA was extracted from cultured astrocytes by using an RNeasy Mini Kit (74104; QIAGEN) according to the manufacturer’s protocols. cDNA was prepared by using a Transcriptor First Strand cDNA Synthesis Kit (04897030001; Roche). The mRNA expression levels were assessed with real-time PCR by using FastStart Essential DNA Green Master (06924204001; Roche). A fragment of Actin was amplified as the internal control. The sequences of the forward (F) and reverse (R) primers used to amplify AMFR, SP1, MAZ, FOXA3, DPF2, and Actin were as follows: AMFR (F), 5′-ATGGAGGCCAGGTTTGCAG-3′, and AMFR (R), 5′-TGCATGTTGGACAGGAGGTG-3′; SP1 (F), 5′-GGGAAACGCTTCACACGTTC-3′, and SP1 (R), 5′-ACCTGGGCCTCCCTTCTTAT-3′; MAZ (F), 5′-AGGACCGCATGAGTTACCAC-3′, and MAZ (R), 5′-AAGCTGCCTCACATTTCTCAC-3′; FOXA3 (F), 5′-AGTGCCTGTAGAGAGACCGA-3′, and FOXA3 (R), 5′-TCACTGGAGAATACACCTCGC-3′; DPF2 (F), 5′-GAGCACGGAAGCGGATCAT-3′, and DPF2 (R), 5′-CATAGGGCTTATCCCGGTCC-3′; Actin (F), 5′-CATGTACGTTGCTATCCAGGC-3′, and Actin (R), 5′-CTCCTTAATGTCACGCACGCACGAT-3′. All reactions were run on a Roche LightCycler 96 real-time PCR instrument. A total of 3 biological replicates and 3 technical replicates were performed for each group. The data were analyzed using the comparative 2^-ΔΔCt^ method.

### Tissue processing, IHC, and analysis.

The mice were deeply anesthetized with pentobarbital sodium and then perfused with 10% formalin. Their brains were rapidly removed, fixed in 10% formalin for 24 hours, and dehydrated with 30% sucrose in PBS. Then, the brains were sectioned with a microtome and mounted on precoated glass slides. The sections were then incubated with anti-GFAP antibody (3670S; Cell Signaling Technology), anti-AMFR antibody (16675-1-AP; Proteintech), or anti-EAAT2 antibody (22515-1-AP; Proteintech) followed by Alexa Fluor 488–conjugated donkey anti-rabbit IgG (A21206; Thermo Fisher Scientific) or Alexa Fluor 594–conjugated donkey anti-mouse IgG (11058; Thermo Fisher Scientific) secondary antibody. DAPI was used as a nuclear counterstain. Images were captured with a confocal laser microscope (880; Zeiss). The amplifier offset and detector gain were adjusted and then left unchanged during each experimental session. To measure AMFR expression in GFAP-labeled astrocytes in the hippocampus, images of the molecular layer of the dentate gyrus were captured at 40× magnification, converted to 8-bit images, and autothresholded using ImageJ software (NIH). The average IF intensity of AMFR in the GFAP-labeled areas was calculated. Qualification was performed using 3 original magnification 40× images of 3 adjacent coronal sections from each mouse brain. A total of 9 data points were obtained from 3 mice per group.

### In vivo AMFR OE or KD.

To induce the expression of AMFR in hippocampal astrocytes, the coding DNA sequence of the mouse AMFR gene (Gene ID: 23802) was synthesized and subcloned into the pAAV-GfaABC1D plasmid vector to construct the AAV5-AMFR virus (Taitool Bioscience). The shRNA was used to achieve AMFR KD. The target sequences were as follows: AMFR-shRNA, 5′-GGACTTCAGTGAGGTAGAA-3′, and negative control shRNA, 5′-CGCTGAGTACTTCGAAATGTC-3′. The selected oligos were then subcloned into the AAV2/5 plasmid vector under the control of the H1 promoter to construct the AAV2/5-H1-shRNA-CAG-EGFP virus (Taitool Bioscience). Recombinant AAV vectors diluted in PBS (5 × 10^12^ virus genome/mL) were bilaterally injected into the hippocampi of 8-week-old *C57BL/6J* mice (anteroposterior = −2.0 mm; mediolateral = ±1.8 mm; dorsoventral = −2.3 mm) in a volume of 500 nL. The mice were subjected to behavioral analysis 4 weeks after AAV injection.

### Acute and chronic models of PTZ-induced seizures.

PTZ (P6500; Sigma-Aldrich) was dissolved in sterilized normal saline. LTG (HY-B0495; MCE) and APAP (HY66005; MCE) were dissolved in DMSO (D8418; Sigma-Aldrich). The prepared reagents were administered in a volume of 0.1 mL through i.p. injection. Acute seizures were induced by a single dose of 55 mg/kg PTZ. The EAAT2 inhibitor DHK (D1064; Santa Cruz Biotechnology), which was i.p. injected at a dose of 10 mg/kg 30 minutes before PTZ, was used to distinguish whether the antiepileptic effect of APAP was due to its ability to increase functional EAAT2 oligomer levels. To study the effects of APAP on epileptogenesis, mice were repeatedly injected with 40 mg/kg PTZ once every other day. APAP or vehicle was administered every day. Epileptic seizures were graded by an observer blinded to the experimental conditions using a 5-point seizure score: 0) no behavioral signs; 1) whisker trembling and/or facial jerking and neck jerks; 2) clonic seizure in a sitting position; 3) tonic-clonic seizure (lying on belly); 4) tonic-clonic seizure (lying on side) or wild jumping.

### KA-induced chronic TLE model and video-EEG analysis.

The KA-induced chronic TLE mouse model was established as previously described. Mice exhibited chronic spontaneous seizures 5 weeks after unilateral intrahippocampal injection of 200 ng KA (K0250; Sigma-Aldrich). Baseline motor seizures were recorded via wired EEG for 14 days before administration of vehicle or APAP. The mice that exhibited epilepsy were randomly divided into 3 groups. There were 7 mice in the DMSO vehicle group, 10 mice in the APAP group, and 6 mice in the APAP+17AAG group. During vehicle or drug administration, 1 mouse in the vehicle group and 2 mice in the APAP group died of lethal seizures. At the end of the experiment, EEG data from 6 vehicle-treated mice, 8 APAP-treated mice, and 6 APAP+17AAG-treated mice were analyzed. The baseline seizure frequency (number of seizures per day) was measured from 14 days of video-EEG data recorded before vehicle or drug administration. Seizure frequency during vehicle or drug treatment was measured from video-EEG data acquired between day 1 and the endpoint. We attempted to record the EEG data for each mouse for as long as possible during drug or vehicle administration (up to 30 days in this study); however, as the contact between the EEG electrode on the EEG head implant and the scalp sometimes became loose in the free-moving mice, we terminated EEG recording and treatment when the EEG signal became noisy. During EEG recording, each mouse was housed in a special cage, and the EEG data were examined in a double-blinded manner to determine the presence of seizures.

### In-cell ELISA.

A total of 2386 FDA-approved drugs were obtained from a compound library (HY-L022; MCE) and a self-built compound library. U2OS cells were used for the in-cell ELISA due to their good adhesion ability. U2OS cells were seeded in 96-well plates. When they reached 80% to 90% confluence, the cells were treated with each drug at a concentration of 2 μM or 10 μM for 24 hours. Then, the cells were fixed in 4% paraformaldehyde for 10 minutes and washed with PBS-Tween 3 times. The cells were blocked and permeabilized using blocking buffer (5% BSA in 0.1% Triton X-100) for 30 minutes at room temperature (RT). The cells were incubated with primary antibodies (AMFR, 1:20,000, 16675-1-AP; Proteintech) diluted in 1% BSA and 0.1% Triton for 3 hours at RT. Thereafter, the cells were incubated with secondary antibody (HRP-conjugated goat anti-rabbit, 1:5000, W4011; Promega) for 1 hour at RT. After 3 additional washes, binding was visualized with 2-component chromogenic TMB (PR1210; Solarbio) for 10 minutes. The reaction was stopped with stop reagent (C1058; Solarbio), and the signal intensity was measured at 450 nm with a FLUO star microplate reader (BMG Labtech) within 15 minutes. Eight DMSO-treated wells were used as controls for each plate. For each compound, treatment was performed in duplicate.

### Statistics.

Prism software 8.2 (GraphPad) was used to calculate statistical significance using unpaired or paired 2-sided Student’s *t* tests, Mann-Whitney *U* tests, or 1-way ANOVA or 2-way ANOVA followed by multiple comparisons testing. All data are presented as the mean ± SD. Significance was determined when the *P* value was less than 0.05.

### Study approval.

All adult animals were housed and handled in accordance with protocols approved by the institutional review board of the IBMS & Peking Union Medical College and were conducted according to the Beijing Administration Office of Laboratory Animals guidelines for the care and use of laboratory animals. The use of the surgically removed hippocampal tissues was approved by the institutional review board of IBMS & CAMS, Beijing, China.

## Author contributions

LS and GL conceived the study, performed most of the experiments, analyzed the data, and wrote the manuscript. XZ, YL, YQ, and YD assisted with the animal experiments. WZ provided technical support for the cellular experiments. All the authors read and discussed the manuscript. QX supervised the entire project.

## Supplementary Material

Supplemental data

Supplemental data set 1

## Version 1 08/08/2022

Electronic publication
