# Supplementary material for: Pharmacological induction of AMFR increases functional EAAT2 oligomer levels and reduces epileptic seizures in mice
Source: JCI Insight. 2022 Aug 8;7(15):e160247. doi: 10.1172/jci.insight.160247 (PMC9462477; doi:10.1172/jci.insight.160247)
Supplement: Supplemental data [file jciinsight-7-160247-s042.pdf]

Supplemental figures

Manuscript: Pharmacological induction of AMFR increases functional EAAT2 oligomer levels and reduces epileptic seizures in mice

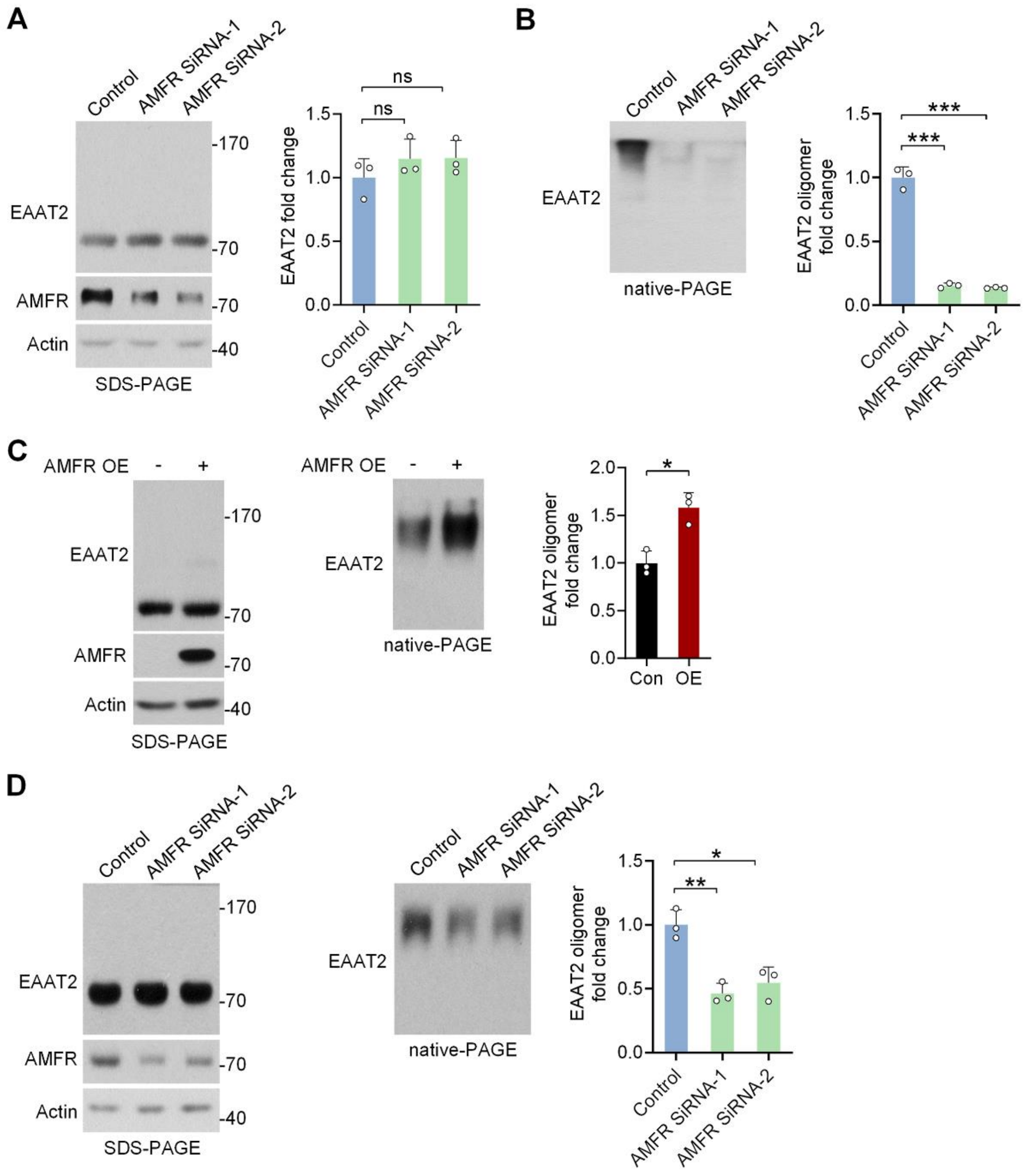

Supplemental figure 1. AMFR expression regulates the level of EAAT2 oligomers. (A, B) SDS–PAGE and native–PAGE followed by immunoblotting analysis of lysates of cultured astrocytes at 48 h posttransfection of two independent siRNAs against mouse AMFR. The intensity of the EAAT2 bands was normalized to that of the actin bands (*n* = 3). (C, D) SDS–PAGE and native–PAGE followed by immunoblotting analysis of lysates of HEK293 cells at 48 h posttransfection of AMFR plasmids or two independent siRNAs against human AMFR. The intensity of the EAAT2 bands was normalized to that of the actin bands (*n* = 3). OE, overexpression. Con, control. One-way ANOVA followed by Dunnett's post-hoc test (A, B, D). Student's t test (C). ns, not significant. * $P < 0.05$, ** $P < 0.01$, *** $P < 0.001$. Data are representative of two or three independent experiments.

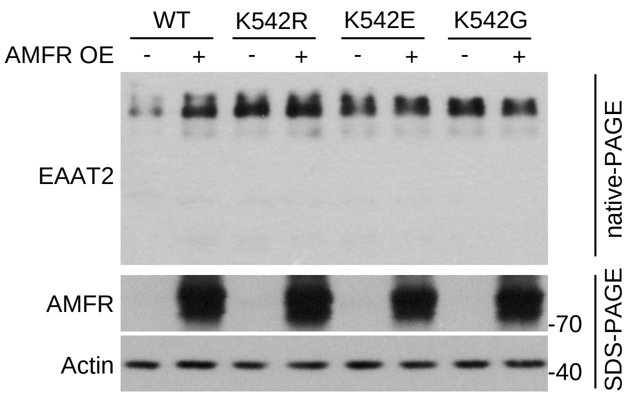

Supplemental figure 2. AMFR regulates the oligomeric state of EAAT2 through ubiquitination at K542. SDS–PAGE and native–PAGE followed by immunoblotting of HEK293 cells at 48 h posttransfection of expression vectors encoding AMFR and WT or EAAT2 mutants at position 542. Empty vector was used as a control for the AMFR vector. Data are representative of two independent experiments.

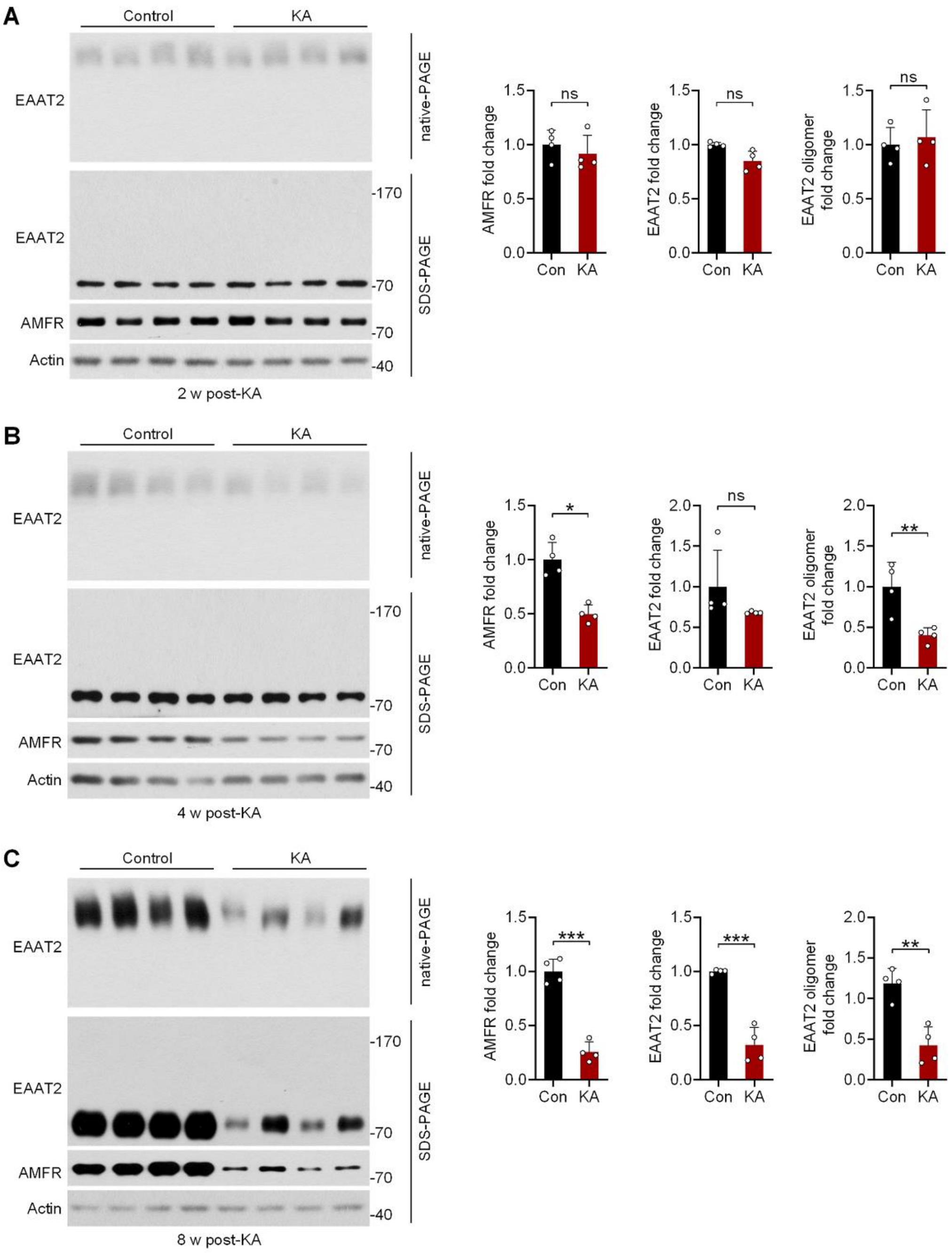

Supplemental figure 3. The levels of total EAAT2, EAAT2 oligomers and AMFR in the hippocampi of mice at different stages after KA injection. (A) SDS–PAGE and native–PAGE followed by immunoblot analysis of lysates from the hippocampi of mice 2 weeks after i.p. injection of KA or vehicle (control). (B) SDS–PAGE and native–PAGE followed by immunoblot analysis of lysates from the hippocampi of mice 4 weeks after i.p. injection of KA or control. (C) SDS–PAGE and native–PAGE followed by immunoblot analysis of lysates from the hippocampi of mice 8 weeks after i.p. injection of KA or control. The bar graph shows the relative fold change in AMFR and total EAAT2 levels determined by SDS–PAGE and EAAT2 oligomer levels determined by native–PAGE. In (A–C), the density of the AMFR and EAAT2 bands was normalized to that of the actin bands (n = 4 per group). Con, control. Student's t test. ns, not significant; * $P < 0.05$, ** $P < 0.01$, *** $P < 0.001$.

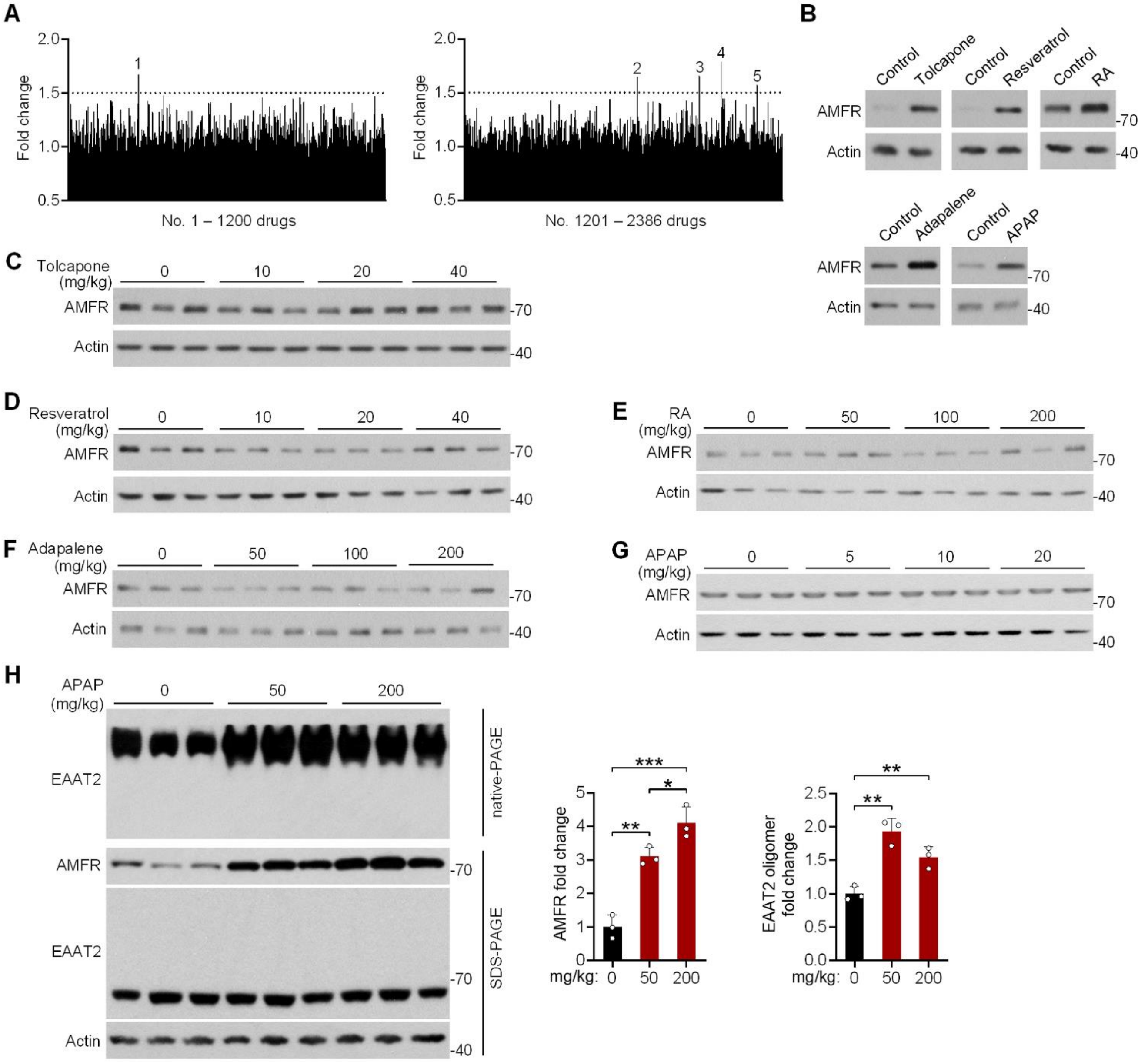

Supplemental figure 4. Screening of compounds that can induce AMFR protein expression. (A) Bar graph showing the change in AMFR levels determined by in-cell ELISA. U2OS cells cultured in 96-well plates were treated with 2,386 FDA-approved drugs for 48 h. Five drugs induced a more than 1.5-fold increase in AMFR levels (1, RA; 2, APAP; 3, adapalene; 4, resveratrol; 5, tolcapone). (B) SDS–PAGE followed by immunoblot analysis of lysates of cultured astrocytes treated with tolcapone, resveratrol, RA, adapalene or APAP at a concentration of 2 μM for 48 h. (C–G) SDS–PAGE followed by immunoblot analysis of lysates from the hippocampi of normal mice after daily i.p. injection of tolcapone, resveratrol, RA, adapalene or APAP at the indicated doses for three days. (H) SDS–PAGE and native–PAGE followed by immunoblot analysis of lysates from the hippocampi of normal mice after daily i.p. injection of APAP at the indicated dose for three days. The density of the AMFR and EAAT2 bands was normalized to that of the actin bands (n = 3). One-way ANOVA followed by Dunnett's post-hoc test. * $P < 0.05$, ** $P < 0.01$, *** $P < 0.001$.

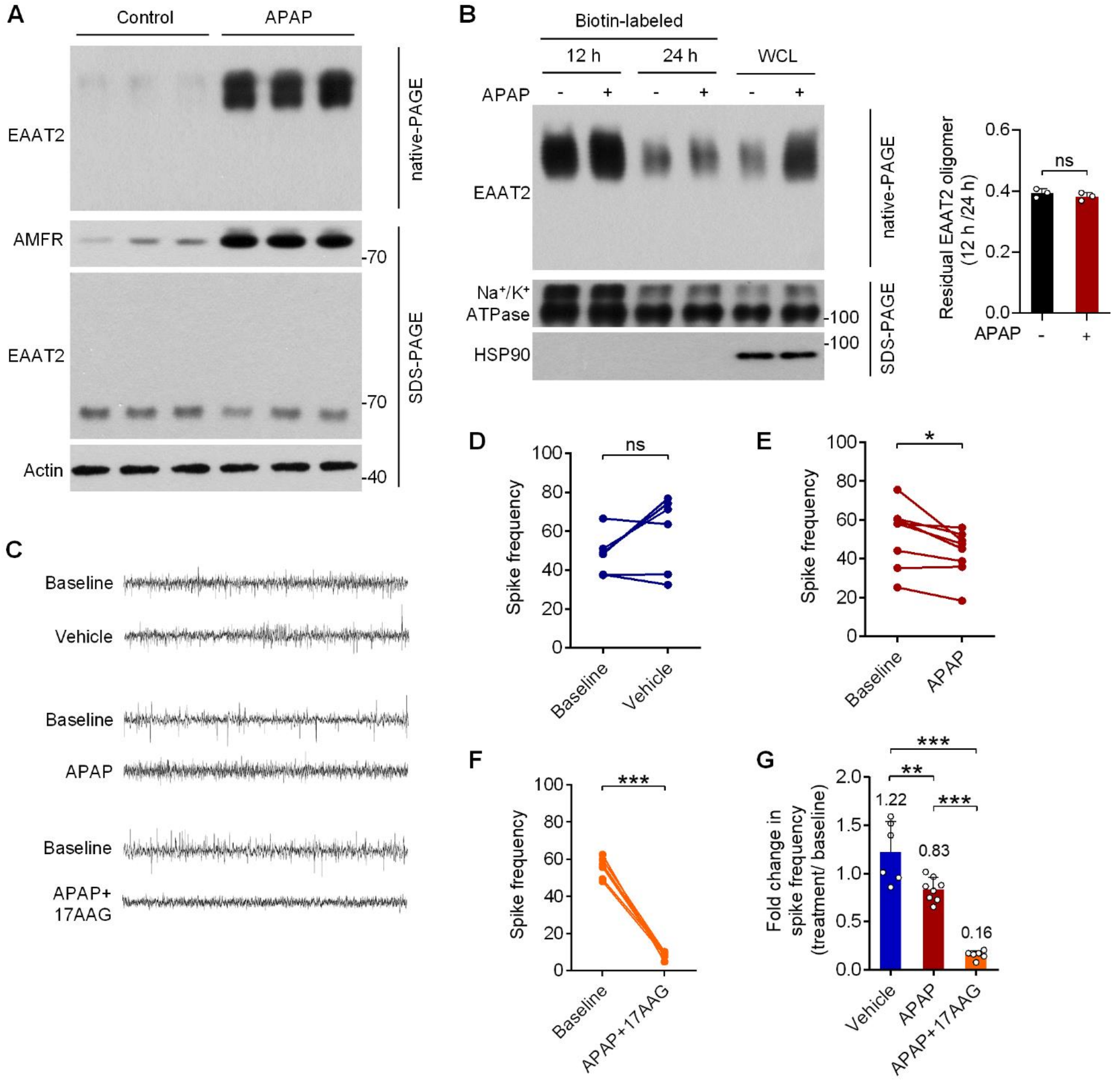

Supplemental figure 5. The antiepileptic effect of APAP in mice with chronic TLE. (A) SDS–PAGE and native–PAGE followed by immunoblot analysis of lysates from the hippocampus ipsilateral to KA injection. Five weeks after hippocampal injection of KA, female mice were given 50 mg/kg APAP or vehicle for 5 days. (B) Pulse–chase analysis. Native–PAGE and SDS–PAGE followed by immunoblotting analysis of lysates of cultured astrocytes in the pulse-chase experiment. Astrocytes were preincubated with sulfo-NHS-SS-biotin for 30 min to label surface proteins and were then treated with vehicle (DMSO) or APAP and collected at the indicated time points post-sulfo-NHS-SS-biotin labeling. The membrane protein Na^+/K^+ ATPase was used to confirm equal loading of proteins. HSP90 was only detected in whole cell lysates (WCL; harvested at 24 h post-APAP treatment) to demonstrate that intracellular proteins were not biotinylated. Student's t test. (C) Representative 1-min EEG tracings at baseline and during treatment with vehicle, APAP or APAP/17AAG. (D–F) Analysis of the difference in the number of interictal epileptic spikes between baseline and after treatment by paired t test. (G) Analysis of the fold change in the number of interictal epileptic spikes before and after treatments by the Mann–Whitney test. ns, not significant.

* $P < 0.05$, ** $P < 0.01$, *** $P < 0.001$.
